# Two endogenous retroviral loci appear to contribute to Multiple Sclerosis

**DOI:** 10.1186/s12883-016-0580-9

**Published:** 2016-04-30

**Authors:** Bjørn A. Nexø, Sara B. Jensen, Kari K. Nissen, Bettina Hansen, Magdalena J. Laska

**Affiliations:** Aarhus University, Biomedicine, Bartholin Building, Wilhelm Meyers Alle 4, DK-8000 Aarhus C, Denmark; Department of Clinical Medicine, Aarhus University, DK-8000 Aarhus C, Denmark; Department of Rheumatology, Aarhus University Hospital, DK-8000 Aarhus C, Denmark; Present address: VIA University College, DK-8200 Aarhus, Denmark

**Keywords:** Endogenous retroviral loci, Synergy of viruses, Multiple sclerosis, Type 1 Diabetes, Rheumatoid arthritis

## Abstract

**Background:**

Two endogenous retroviral loci seem to be involved in the human disease Multiple sclerosis (MS).

**Results:**

The two retroviral loci synergize in and contribute to MS (shown by ANOVA). Synergy probably means recombination or complementation of the activated viruses. Similar observations may be true for Type 1 Diabetes and Rheumatoid arthritis. In MS the genes also synergize with the immune system; this could well be a common phenomenon.

**Conclusion:**

We formulate various theories about the role of the viruses. Also, the concept is developing that some forms of autoimmunity should be treatable with antiretrovirals. In the case of MS, this idea is gradually gaining weight.

## Background

Accumulating genetic evidence as well as studies of viral expression suggests that endogenous retroviruses play a role in MS [1, 2]. We have shown that markers in or near two endogenous retroviral loci, HERV-Fc1 on chromosome X and HERV-K13 on chromosome 19, are associated with disease and synergized in influencing the risk of MS. For synergy, we recoded the cases as 1 and controls as 0. Then, synergy was measured as the p-value of the product term in an ANOVA (*p* = 0.0009) [3]. Thus, we measured multiplicative synergy and not simply additivity. The odds-ratios for the double homozygotes were OR = 0.6 for rs318138^AA^rs2435031^GG^; OR = 1.3 for rs318138^GG^rs2435031^GG^; OR = 1 for the reference genotype rs318138^GG^rs2435031^TT^ and OR = 11 for the risk genotype rs318138^AA^rs2435031^TT^.

Also, the two viral loci synergized with a locus in the major histocompatibility complex, MHC, known to influence MS [3](*p* = 0.02). The most extreme genotype rs318138^AA^rs2435031^GT^rs3135388^CT^ had an OR = 38, even though we applied continuity correction (i.e added 0.5 to all numbers) in order to compensate for small numbers.

Finally, genes for three pattern recognizers for retroviruses in the innate immune system, *TRIM5*, *TRIM22 and BST-2,* influenced the risk of getting MS [4]. Importantly, the two viral genes again synergized with *TRIM5* (Nexø BA et al, unpublished). Thus, there is a reason to believe that the retroviral loci influence the risk of getting MS through the innate immune system, and possibly also through the adaptive immune system. The TRIM5 gene product responds to entry of viral particles into cells. Therefore, we have reason to believe that virus replicates in the patients at least periodically. In accordance with this, expression of the two viruses was increased in lymphocytes and plasma in relation to MS [5] (Laska MJ et al, unpublished). It must be emphasized that these results are for the common forms of MS, relapsing/remitting MS and secondary progressive MS, while different mechanisms may apply to the rarer primary progressive MS [6]. Also, bear in mind that we are mainly studying markers on the chromosomes, and nearby genes (< approximately 40 kb) could distort the results. However, the presence of synergy is an argument for a role of the viral loci.

Similar, although less extensive, observations have been made in two other autoimmune diseases: Type 1 Diabetes (T1D) and Rheumatoid Arthritis (RA) [3]. In both diseases, a set of retroviral loci appeared to contribute to the risk of disease and also synergized in this effect. Notably, a separate set of retroviral loci contributed to each of the three diseases. MS was mentioned above. In T1D, the viruses were HERV-K106 and HERV-K119 on chromosome 3 and 12, respectively. In RA, it was a HERV-K on chromosome 22 and a HERV-H, also on chromosome 22. In addition, HERV-Fc1 on chromosome X seemed to contribute to RA. All these observations were initially made in fairly focused Scandinavian populations. However, the association of HERV-Fc1 with MS has been repeated in two out of three Spanish populations [7]. The population, which failed to show the association, was the southernmost, i.e. from the region of Spain, where non-European ethnic influences are stronger. Thus, there may well be ethnic issues relating to the above findings. Along the same line: In Spain, it appears that a HERV-K on chromosome 1 is associated with MS [8]. We do not know if it replaces the one observed on chromosome 19 in our studies of Scandinavians.

These observations may well go beyond autoimmune diseases: We have observed a synergy of two viral loci, HERV-Fc1 on the X chromosome and HERV-K on chromosome 1 in the mature plasma B-cell neoplasm Multiple Myeloma (MM) [9]. These findings could again indicate viral recombination or complementation. In chickens and mice, replicating retroviruses cause leukemia by integrating into and upregulating growth promoting regions of the chromosomes [10, 11]. It is tempting to speculate that similar mechanisms apply in human leukemia and autoimmunity. So maybe an important part of autoimmunity is viral lesions in and clonal expansions of certain immune cells. Also, the oligoclonal IgG bands that are observed in MS intrathecal fluid might then be explained as the result of cells with viral lesions [12]. This represents an alternative, immediately testable hypothesis for viral action in MS and other autoimmune diseases. Under this hypothesis, we see two possibilities: The virus activates a dormant autoimmune cell, or it activates a primitive lymphoid cell and develop its antigen specificity, more or less at random. Importantly, a viral transformation of lymphocytes may also be tested in animal studies. Lake Casitas virus is a feral mouse retrovirus that causes hind leg paralysis in laboratory strains of mice [13].

### Why endogenous retroviruses?

Why do retroviral loci play a role in autoimmunity? Endogenous retroviruses span the divide between self and foreign. On one hand, they are latently present in all nucleated cells of the body, and there is no way the immune system can rid the body of them. On the other hand, they still have the ability, if expressed, to activate genes and/or to stimulate the innate immune system and trigger a response like a foreign microorganism. And even in situations where the viruses are not endogenous but horizontally transmitted retroviral infections have great permanence and generally last for life. So we believe that many forms of human autoimmunity may be influenced by retroviruses. A well-known example is human Tropical Spastic Paraparesis, which is caused by a human T-lymphotropic virus, HTLV [14]. However, retroviruses may not be the only agents having this effect. Other microorganisms establish intimate, long lasting relationships with the human body, most notably the bacteria of the gut.

Why sets of retroviral loci in autoimmunity? It seems that no retroviral locus in the genome is complete in the human populations for which there are sequences. Of the approximately 100,000 retroviral sequences only 51 can with zero to two mutations encode a viral protein [3]. So defined, a few loci encode all three genes, but zero to two breaks in the reading frame may be present, and there may also be point mutations. Thus, we think recombination or complementation between at least two viruses is necessary for infectious viral particles to form. Recombination of viral information could take place by formation of mixed particles containing two different viral genomes, i.e. during complementation [15]. In successive steps, information from more than two viral sequences may be recruited. Thus, we by no means exclude the contribution of more than two loci. However, the present group-sizes investigated (about a thousand persons or less) and techniques applied are probably not sufficient to demonstrate a 3-way synergy between viruses. In Rheumatoid Arthritis, we have seen suggestive evidence that three viruses contribute, but no formal proof. The issue may be resolved if we obtain sequences of recombined viruses.

### Comparison of diseases; Contributions of viruses

Retroviruses, in general, have three genes, *gag, pol* and *env,* and a noncoding region, the LTR, which contains regulatory elements such as a promoter and an enhancer. The locus HERV-Fc1 may contribute to recombination or complementation in MS, RA and MM. In all three situations, we think the locus could provide the ENV function. HERV-Fc1 *env* seems intact (Jensen SB et al, unpublished observation). Moreover, HERV-Fc1 is a gamma-retrovirus, and the ENV of these viruses has the advantage that the surface protein is covalently linked to transmembrane protein by an S-S bridge [16]. Recruitments of *env* from gamma-retroviruses to beta-retroviruses have been seen before [16]. In contrast, sequence analyses show that HERV-Fc1 *pol* is interrupted in several places and that HERV-Fc1 GAG does not contain the usual processing sites phe-pro or tyr-pro. In accordance with this, the HERV-Fc1 GAG polyprotein is not properly processed but remains a polyprotein when human cells are transfected with expression systems containing HERV-Fc1 *gag* and/or *gagpol* (Nissen KK, et al., unpublished observation). Reverse transcriptase activity was also not detectable in pelleted supernatants from such cells.

The other locus observed in MS is a HERV-K locus on chromosome 19. All except three viral loci with intact or near-intact *pol* regions in the human genome belong to this family. HERV-Ks are the only candidates for contributing the POL functions in the four diseases here. This is important as POL functions, protease, reverse transcriptase, and integrase are the main targets for drug action in antiretroviral therapy. This means that an attempt at treating autoimmune diseases with antiretrovirals could usefully draw on the drug-sensitivities of HERV-Ks for drug selection. Several papers suggest that MS is suppressed in HIV patients treated with a cocktail of antiretrovirals [17–19] but not in untreated HIV [19, 20].

HERV-Ks may further contribute the GAG function in MS, MM, T1D and possibly RA. However, HERV-H on chromosome 22 could also contribute the GAG function in RA. It is presently unclear, which loci could potentially provide the LTRs.

Could increased complexity of the viral interactions translate into the later debut of the disease? In Type 1 Diabetes we observed the interaction of two similar viruses. This disease often has a childhood debut. In MS, we have observed interaction between two dissimilar retroviruses. This disease typically starts in patients in their twenties or thirties. In Rheumatoid Arthritis, we may see interaction of 3 dissimilar viruses. This disease typically unveils at age fifty or sixty. Thus, in this limited material a tendency is present. It would be most interesting to investigate a related disease, Juvenile Idiopathic Arthritis, which typically debuts very early, sometimes at an age of 1 to 2 years. Is this a retrovirally related disease, too, and if so, how do the virus(es) activate so quickly? Could these patients carry a complete viral locus?

### Theories about MS

There are two contemporary sets of data for genetic effects in MS: The outcome could be a result of many minor variations in a multitude of genes [21]. Sawcer et al. analyzed thousands of DNA from cases and controls recruited in Europe and North America utilizing Illumina Chips, which analyze hundreds of thousands of SNPs, and used sophisticated modelling to compensate for different ethnicity. The authors concluded that in the order of a hundred genes influence the risk of MS, many of them genes in the immune system.

Alternatively, MS could be the result of a few drastic changes (this paper). For several reasons, we favor the second possibility. There is a lot of slack in biological systems, and they are able to accommodate small perturbations gracefully. Of course, one can invoke chaos theories, but biological systems, in general, are not chaotic, but highly structured. Random molecular processes occur but unless they affect the genome they do not tend to produce basic instability. We think a fundamental change in the system is necessary, for instance by mutation, viral invasion, or pharmaceutic or toxic drugs. This leads naturally to a few important genes.

On a technical level there is no disagreement. We have purposely concentrated on retroviral loci. Sawcer et al. used Illumina chips. These chips were deliberately designed to focus on single-copy coding genes, and have a scant representation of multi-copy sequences, such as retroviruses. Thus, Sawcer et al. could easily have overlooked a retroviral influence. Moreover, they did not investigate SNPs on the X chromosome, and this left out HERV-Fc1.

Result-wise, we observed ten to forty-fold higher odds-ratios than Sawcer et al. did, but they investigated considerably more cases and obtained lower p-values. Both sets of results could be true. One could, for instance, speculate that the MS-associated variations in the many immune genes described in [21] increase the level of surviving autoimmune cells, and that activated retroviruses transform one or more of these to proliferating clone(s).

There is a large body of literature describing the presence of retroviral particles, called MSRV and related to HERV-W in MS patients (see for instance [22] and quotations thereof). Unfortunately, these investigators generally based their research on proving Koch’s postulates for the MSRV. However, the endogenous retroviruses are always present in the human body, at least in latent form, and their presence, therefore, does not signify a contagious event. More importantly, the definitive Koch experiment: To isolate the agents and infect humans with the purified strains to cause disease cannot be done, as it is unethical. In short, Koch’s postulates cannot be applied to human endogenous retroviruses. Other routes, such as the genetic, may be more productive. If one insists on reconciliation, we study the role of viral proteins, whereas Perron et al. study the viral RNA.

## Conclusions

The results presented here are all fairly new, and much remains to be investigated. We are particularly looking forward to other scientific groups making additional findings or confirming our results.

### Ethics approval and consent

All involved patients gave written and oral consent. Studies of MS, RA, and T1D were approved by the Mid-Jutland Committee for Science Ethics. The study of MM was approved by Committee for Science Ethics in Copenhagen.

### Consent for publication

This paper contains no individual person data.

### Availability of data and material

These will not be made available here as this is a review and primary data are not presented.

## Abbreviations

MS: multiple sclerosis
MHC: major histocompatibility complex
T1D: Type 1 Diabetes mellitus
RA: Rheumatoid arthritis
MM: Multiple myeloma
HERV: human endogenous retrovirus
HIV: Human immunodeficiency virus
HTLV: human T-cell lymphotropic virus
Gag: retroviral gene encoding the core particle
Pol: retroviral gene encoding the enzymes of the virus
Env: retroviral gene encoding the envelope proteins
LTR: Long terminal repeat, encoding retroviral promoter and enhancer
